# Effect of double disinfectant application during premilking teat disinfection on teat cleanliness, bacterial count, and mastitis in pasture-grazed dairy cows

**DOI:** 10.3168/jdsc.2024-0696

**Published:** 2025-01-10

**Authors:** Thiago Resin Niero, Roberto Kappes, Angelica Leticia Scheid, Andreina Ferreira Ramos, Larissa Henrique da Silva, Leonardo Leite Cardozo, Sandra Maria Ferraz, André Thaler Neto

**Affiliations:** 1 Centro de Ciências Agroveterinárias, Universidade do Estado de Santa Catarina (CAV/UDESC), Lages, SC, Brazil 88520-000; 2 Ordemilk, Treze Tílias, SC, Brazil 89650-000

## Abstract

The use of double disinfectant application during premilking teat disinfection on heavily soiled teats has proven effective in reducing the teat cleanliness score and certain bacterial groups in freestall confined cows. We aimed to compare the efficacy of a single (SDA; before forestripping) and a double (DDA; before and after forestripping) disinfectant application on teat cleanliness score, bacterial counts, and the incidence of subclinical and clinical mastitis in pasture-grazed dairy cows. Initially, 2 groups of 8 cows were assigned to receive either SDA or DDA lactic acid and hydrogen peroxide application, using a dip cup. The treatments were applied at every milking to all lactating cows participating in the experiment. The experiment was conducted over an 8-mo period. Cows calving during this period were alternately assigned to one of the 2 groups after 7 d. Every 15 d, both before (PRE) and after (POST) treatment, we evaluated the teat cleanliness score (TCS) and collected swabs from teat skin to quantify gram-negative bacteria (coliform and noncoliform), *Staphylococcus* spp., *Streptococcus* spp., and total bacterial count (TBC). On the same day, we also collected milk samples from individual cows to measure SCC and identify subclinical mastitis (SCC ≥200,000 cells/mL). We then classified the cows as having no subclinical mastitis and new subclinical cases based on 2 consecutive analyses. Clinical mastitis cases were recorded throughout the experiment. A variance analysis was performed to evaluate the effect of treatment on bacterial count, TCS, and SCS. After disinfection, gram-negative and *Streptococcus* spp. were the most reduced type of bacteria regardless treatment. No significant differences were observed between the DDA and SDA groups for TCS, counts of coliforms, gram-negative noncoliform bacteria, *Staphylococcus* spp., *Streptococcus* spp., and TBC PRE and POST application or in reducing these variables. We transformed SCC into SCS, which was similar between groups, as well as the subclinical and clinical mastitis cases. In situations with lightly soiled teats, an extra application of disinfectant during premilking teat disinfection did not significantly improve TCS, reduce bacterial counts, SCS, or decrease the incidence of subclinical and clinical mastitis compared with a single application of disinfectant.

## Highlights

• Environmental conditions are conducive to lightly soiled teats before milking.
• DDA is not necessary when cows have lightly soiled teats.
• Clinical and subclinical mastitis dynamics were similar between treatments.

## Graphical Abstract

**Figure:**
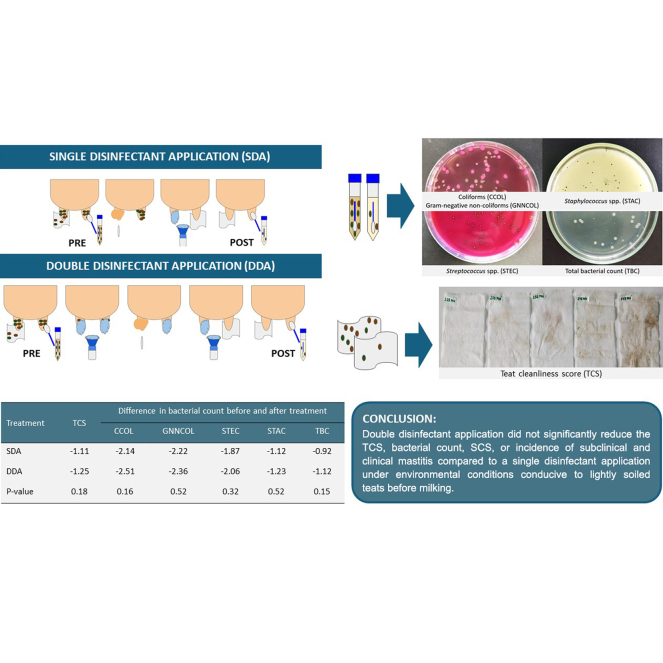
**Summary:** This study compares the efficacy of a single disinfectant application (SDA) and double disinfectant application (DDA) during premilking teat disinfection in relation to teat cleanliness, bacterial counts, and the incidence of subclinical and clinical mastitis in pasture-grazed dairy cows. On average, the cows had lightly dirty teats, leading to no significant difference in teat cleanliness scores and bacterial count reductions between the 2 treatments. The dynamics of clinical and subclinical mastitis were also similar between the 2 groups.

Environmental conditions, such as rainfall and high relative humidity, are known to influence udder and teat cleanliness, especially in pasture-grazed dairy herds. In these systems, cows are more exposed to mud depending on the conditions of paddocks and tracks, which can increase teat contamination by environmental microorganisms (Arnott et al., 2017; Morton et al., 2014; Nogara et al., 2022). The cleanliness score of teats and the initial level of contamination influence the effectiveness of premilking procedures (Gleeson et al., 2009; Córdova et al., 2018). Poor hygiene of dairy cows and increased teat soiling have consistently been associated with higher SCC and an elevated risk of subclinical udder infections (Reneau et al., 2005; Kappes et al., 2020). Effective premilking teat sanitation reduces both the soil and bacterial load on teat skin, thereby decreasing milk contamination and preventing intramammary infections by reducing the influx of pathogens through the teat canal (Bramley and McKinnon, 1990; Baumberger et al., 2016).

To improve teat cleanliness and reduce bacterial counts, manual washing and drying of teats with water (Gleeson et al., 2009) or washing before disinfectant application can be effective methods. However, these approaches have limitations, including increased water use, the need to dry the entire wetted surface, and added time required for management (Gibson et al., 2008). Alternatively, an additional application of disinfectant may be used to moisten the teat, facilitating dirt removal along with reducing the time needed for the procedure. This was observed in a previous study in confined cows, where applying an additional disinfection during premilking teat disinfection on heavily soiled teats showed to be more effective in reducing the teat cleanliness score, *Staphylococcus* spp. count, and total bacterial count (**TBC**) compared with a single application of disinfectant (Niero et al., 2024).

Our hypothesis was that forestripping with disinfectant-moistened teats, followed by reapplication of disinfectant, would be more effective in reducing teat soiling and bacterial count, compared with dry teats, due to the prolonged disinfectant contact time, which softens the soil, removing it more easily and thereby decreasing the incidence of clinical and subclinical mastitis. Additionally, the disinfection of milkers' gloves during forestripping in double disinfectant applications can reduce the bacterial count (Niero et al., 2024). Our objective was to compare the effect of a single disinfectant application (**SDA**) with a double disinfectant application (**DDA**) during premilking teat preparation on teat cleanliness score (**TCS**), bacterial counts, and the incidence of subclinical and clinical mastitis in pasture-grazed dairy cows.

This research was conducted in the dairy cattle unit of the University of Santa Catarina State (UDESC–CAV; Lages, Brazil), in southern Brazil (latitude: 27°48′58″, longitude: 50°19′34″, altitude 950 m above sea level). The region is located in a subtropical humid climatic zone, type Cfb according to the Köppen classification, with rainfall regularly distributed throughout the year (Köppen and Geiger, 1928). The experiment was conducted from November 2021 to July 2022. The cows were milked twice a day at 0700 and 1500 h in a herringbone parlor using a milking system with automatic clusters removers. After milking, each cow received 3 kg of concentrate in the feeding area (6 kg/cow per day) and then had free access to pasture until the next milking. The herd was composed of Holstein and crossbred Holstein × Jersey cows.

Before the experiment began, 16 cows were selected addressing the following 2 criteria: DIM ranging from 7 to 215, and no clinical mastitis in the previous month. These 16 cows were divided into 2 homogeneous groups based on breed, parity, DIM, milk yield, SCC, teat-end hyperkeratosis score, udder depth, and udder clearance, to ensure similarity between these 2 groups for these traits. Teat-end hyperkeratosis was classified on a scale from 1 to 4, where 1 = no ring formation, 2 = small ring formation, 3 = rough ring formation, and 4 = very rough ring formation (Mein et al., 2001). Udder depth was measured in centimeters as the distance from the udder floor to the hock line, and udder clearance was measured as the distance from the udder bottom to the ground before milking, as described by Knob et al. (2020).

After the experiment began, additional cows in the herd that calved during the experimental period were alternately assigned to one of the 2 groups after 7 DIM. In total, 4 cows were assigned to the SDA group and 5 to the DDA group throughout the experiment. Overall, the SDA group consisted of 12 cows and the DDA group of 13 cows. During the same period, 5 cows from the SDA group and 7 from the DDA group were dried off.

The groups formed at the beginning of the experiment had similar averages for parity (2 for both), milk yield (23.8 and 21.9 kg/d), DIM (132 and 131 d), SCC (93,000 and 96,000 cells/mL), teat-end hyperkeratosis score (2.0 and 1.5), udder depth (14.5 and 15.0 cm above the hock), and udder clearance (62.0 and 65.5 cm above the ground) for SDA and DDA groups, respectively.

The SDA treatment consisted of (1) forestripping 3 streams of milk per quarter, (2) applying a disinfectant based on lactic acid and hydrogen peroxide (Peroxilac, Weizur) using a dip cup, (3) allowing at least 30 s of contact time, (4) drying with a paper towel per teat, and (5) attaching the milking unit. The DDA treatment consisted of (1) applying a disinfectant based on lactic acid and hydrogen peroxide (Peroxilac, Weizur) using a dip cup, (2) forestripping 3 streams of milk per quarter, (3) applying the same disinfectant, (4) allowing at least 30 s of contact time, (5) drying with a paper towel per teat, and (6) attaching the milking unit. Premilking procedures were performed on all cows daily throughout the 8-mo duration of the experiment. Postmilking disinfection was the same for both groups, entailing immersion of the teats in an iodine-based disinfectant (Blocking, Weizur). The cows remained in the same environment for the duration of the experiment, with each group identified by a different color tape on their feet.

The TCS was assessed rubbing a moist towel 3 times from top to bottom of the left fore teat before treatment and on the right rear teat after treatment. The towels were identified with the cows' numbers and labeled as either before (**PRE**) or after (**POST**) treatment. The towels were air-dried at room temperature and visually categorized into 5 cleanliness scores (0 = clean, 1 = almost clean, 2 = lightly dirty, 3 = dirty, and 4 = extremely dirty) based on the area and thickness of the dirt, as described by Hovinen (2009).

Before premilking routine, a teat skin sterile swab was collected from the cranial surface of the right fore and left rear teats (PRE) from top to bottom, using the same swab for both teats. After the premilking routine was completed (before unit attachment), a new sterile swab was collected from the external side of the same teats (POST) from top to bottom, using the same swab for both teats. Before collection, the swabs were moistened in buffered peptone water (Oxoid Ltd., Basingstoke, UK). After sample collection, the swabs were placed in 4-mL tubes with buffered peptone water and refrigerated for subsequent analysis on the same day. The samples were vigorously vortexed, and serial dilutions were performed to identify and enumerate the colonies. For PRE samples, serial dilutions of 1:10, 1:100, and 1:1,000 were prepared, whereas POST samples were diluted 1:10 or undiluted. For the 1:10 dilution, 1 mL of the sample was transferred into a sterile tube with 9 mL of sterile saline solution. For the 1:100 dilution, 1 mL of the 1:10 solution was transferred into a new sterile tube with 9 mL of sterile saline solution. The same process was repeated once more for the 1:1,000 dilution.

The bacterial count considered coliforms, gram-negative noncoliforms, *Staphylococcus* spp., *Streptococcus* spp., and TBC. MacConkey agar (Oxoid Ltd., Basingstoke, UK) was used for enumerating coliform (lactose-fermenting in red or pink color) and gram-negative noncoliform bacteria (colorless colonies). The total count of gram-negative bacteria corresponded to the sum of coliforms and gram-negative noncoliforms. *Staphylococcus* spp. was counted on Baird–Parker agar (Oxoid Ltd., Basingstoke, UK) enriched with 5% tellurite egg yolk (Newprov, Pinhais, Brazil). *Streptococcus* spp. was counted on modified Edwards agar (Oxoid Ltd., Basingstoke, UK) supplemented with 5% sterile bovine blood. Total bacterial count was determined using plate count agar (**PCA**; Kasvi, São José dos Pinhais, Brazil).

We used 1:100 and 1:10 dilutions for PRE and POST samples, respectively. The PRE and POST dilutions were tested in a pilot study to determine the optimal dilution for bacterial growth and allow for accurate colony counting. For each sample of PRE and POST, we plated one inoculum of 100 µL onto the Baird–Parker and Modified Edwards plates, one inoculum of 50 µL onto MacConkey, and one inoculum of 10 µL onto PCA plates. The inoculum was spread on the plate using a Drigalski spatula. The plates were incubated at 37 ± 1°C for 24 h to count the colonies. For bacterial groups with a null count in the PRE samples, no count was performed in the POST samples for these bacterial groups. Overall, 174 samples showed no bacterial growth. Colony-forming units per milliliter were calculated by multiplying the number of colonies, the dilution factor, and the correction factor by milliliters. The correction factor was 10 for Baird–Parker and modified Edwards, 50 for MacConkey, and 100 for PCA.

An individual composite milk sample from all morning and afternoon milkings was collected using Waikato milk meters (Waikato Milking Systems, Hamilton, NZ) to determine the SCC using flow cytometry. The cows were classified based on SCC, according to Schukken et al. (2003), as follows: no subclinical mastitis (SCC <200 × 10^3^ cells/mL in 2 subsequent analyses) and new subclinical mastitis (SCC <200 × 10^3^ cells/mL in the first analysis and SCC ≥200 × 10^3^ cells/mL in the second analysis). Clinical mastitis was diagnosed by visible milk alterations. When the interval between 2 clinical infections in the same quarter was longer than 14 d, it was considered a new clinical case.

Every 15 d during the morning milking, the same person evaluated the TCS and teat skin bacterial count PRE and POST treatment. We also collected milk samples to evaluate the SCC. In total, 18 d of samples collection were performed.

A post-study sample size calculation was performed using the GPower software (version 3.1.9.7; https://www.psychologie.hhu.de/arbeitsgruppen/allgemeine-psychologie-und-arbeitspsychologie/gpower). A medium Cohen's effect size (d = 0.5; Cohen, 1988), combined with a power (1 − β) of 0.8, an α error of 0.05, 2 groups, and 18 measurements, resulted in a sample size of n = 34. To assess the normality of residuals, the teat bacterial count were transformed into the log_10_ and the SCC was transformed into the SCS (calculated as SCS = log^2^ (SCC/100) + 3; Ali and Shook 1980). The data of teat bacterial count, TCS, and SCS were analyzed by ANOVA with cows with repeated measures over time in each cow, using the MIXED procedure from SAS statistical software 9.4 (SAS Institute Inc., Cary, NC). The statistical model included the effects of the disinfectant application method (SDA or DDA), the day of sample collection, DIM, parity, and the interactions between disinfectant application method and the other variables. As no significant interaction between these variables were detected, the interactions were excluded from the model. The variables of teat bacterial count and TCS were analyzed for PRE and POST treatment, as well as the reduction of these values in log_10_. Negative values in Table 1 indicate a reduction in the teat bacterial count and TCS. Statistical differences were defined at the 5% level. The data were previously tested for normality of residuals using the UNIVARIATE procedure of SAS, with the Kolmogorov–Smirnov test, with all the variables showing normality of residuals (*P* > 0.05). The effects of the treatment on the percentage of no subclinical mastitis and new subclinical mastitis, as well as clinical mastitis, were analyzed using the chi-squared test with the FREQ procedure in SAS.

**Table 1:** Mean values ± SD for teat cleanliness score (TCS), teat bacterial count before (PRE) and after (POST) treatment, and *P*-values for a single (SDA) and double disinfectant application (DDA) during premilking teat disinfection

Variable	N	Treatment		*P*-value
		SDA	DDA	
Teat cleanliness score				
TCS_PRE	277	2.13 ± 0.12	2.16 ± 0.12	0.84
TCS_POST	277	1.00 ± 0.08	0.92 ± 0.09	0.41
Dif_TCS	277	−1.11 ± 0.08	−1.25 ± 0.10	0.18
Teat bacterial count^1^				
COLC_PRE	160	3.25 ± 0.15	3.21 ± 0.17	0.86
COLC_POST	160	1.10 ± 0.19	0.67 ± 0.22	0.08
Dif_COLC	160	−2.14 ± 0.21	−2.51 ± 0.24	0.16
GNNCOLC_PRE	194	3.56 ± 0.13	3.59 ± 0.15	0.85
GNNCOLC_POST	194	1.32 ± 0.18	1.21 ± 0.21	0.67
Dif_GNNCOLC	194	−2.22 ± 0.15	−2.36 ± 0.18	0.52
STEC_PRE	254	3.23 ± 0.17	3.34 ± 0.15	0.56
STEC_POST	254	1.27 ± 0.18	1.15 ± 0.22	0.59
Dif_STEC	254	−1.87 ± 0.14	−2.06 ± 0.17	0.32
STAC_PRE	257	4.22 ± 0.17	4.27 ± 0.19	0.76
STAC_POST	257	2.95 ± 0.22	3.18 ± 0.25	0.40
Dif_STAC	257	−1.12 ± 0.13	−1.23 ± 0.15	0.52
TBC_PRE	269	4.70 ± 0.13	4.90 ± 0.14	0.18
TBC_POST	269	3.72 ± 0.10	3.75 ± 0.12	0.79
Dif_TBC	269	−0.92 ± 0.11	−1.12 ± 0.13	0.15

1 All values are expressed as log_10_ cfu per mL. COLC = coliform count; GNNCOLC = gram-negative noncoliform count; STEC = *Streptococcus* spp. count; STAC = *Staphylococcus* spp. count; TBC = total bacterial count; Dif = difference between count before and after treatment. A negative value indicates a reduction.

For the TCS, we recorded 277 observations: 136 for SDA and 141 for DDA. According to the score classification, the teats were lightly dirty PRE for both groups, with an average score of 2.1 (*P* = 0.84; Table 1), decreasing to ∼1 point in both groups POST, with no significant difference between SDA and DDA (*P* = 0.41; Table 1). Consequently, the reduction in TCS was similar between treatments, being 1.11 and 1.25 for SDA and DDA, respectively (*P* = 0.18). The low TCS PRE could be attributed to the low average rainfall, as only 3 out of the 18 sampling days had precipitation exceeding 10 mm. Morton et al. (2014) observed that during periods of below-average rainfall, cows in pasture systems have cleaner and drier teats than usual. In our study, because the teats were lightly dirty before the treatments, we could not observe a substantial reduction in TCS. In another study evaluating freestall confined cows, we observed higher TCS PRE (mean = 3.35) and average reductions exceeding 2 points for both SDA and DDA (Niero et al., 2024).

In total, we collected 295 teat swab samples (143 from the SDA and 152 from the DDA). We did not observe any differences between SDA and DDA in any bacterial group evaluated at PRE, POST, or in its reduction (Table 1). This differs from our previous findings (Niero et al., 2024), where we observed a greater reduction for *Staphylococcus* spp. (*P* = 0.04) and TBC (*P* = 0.01) using DDA with one (40% lactic acid) or a second different disinfectant (40% lactic acid and 1,500 mg/L chlorine-based). A second application of disinfectant extends the contact time with the teats, which, combined with the removal of debris during forestripping on disinfectant-moistened teats, likely enhances its efficacy in reducing microorganism counts, especially on dirtier teats (Niero et al., 2024).

In the PRE evaluations, *Staphylococcus* spp. was the most prominent bacterial group recovered on swabs (4.22 ± 0.17 and 4.27 ± 0.19 log_10_) for SDA and DDA, respectively. Coliforms showed the lowest abundance (3.25 ± 0.15 and 3.21 ± 0.17 log_10_), followed by *Streptococcus* spp. (3.23 ± 0.17 and 3.34 ± 0.15 log_10_) and gram-negative noncoliforms (3.56 ± 0.13 and 3.59 ± 0.15 log_10_) for SDA and DDA, respectively. Consistent with previous research, TBC, *Staphylococcus* spp., and *Streptococcus* spp., counts were generally higher, whereas coliforms and gram-negative noncoliforms presented lower counts in PRE samples from confined cows (Baumberger et al., 2016; Fitzpatrick et al., 2019, 2021; Niero et al., 2024). The greatest numerical reductions were observed for coliforms, gram-negative noncoliforms, and *Streptococcus* spp., with an average decrease of 2.32, 2.29, and 1.96 log_10_, respectively, for both treatments. In previous studies, we found similar reductions for coliforms and gram-negative noncoliforms (Niero et al., 2024). In contrast, *Staphylococcus* spp. and TBC showed the smallest reductions (1.17 and 1.02 log_10_, respectively). Fitzpatrick et al. (2021), employing a combination of lactic acid and hydrogen peroxide, similar to our study, found a greater reduction (*P* < 0.05) for *Streptococcus* spp. compared with *Staphylococcus* spp. (89.9% vs. 59.4%, respectively), though coliforms were not considered in their analysis. The variable reduction rates observed for these microorganisms may be attributed to the type and concentration of disinfectant used. Baumberger et al. (2016) observed different reductions among different microorganisms with increasing concentrations of chlorine dioxide as a disinfectant. For each 100 μL/L increment in chlorine dioxide concentration, the reduction was most pronounced for gram-negative bacteria (0.33 log units), followed by *Staphylococcus* spp. (0.18 log units), *Streptococcus* spp. (0.16 log units), and TBC (0.09 log units; Baumberger et al., 2016). The effectiveness of microbial reduction is complex and influenced by multiple factors, including the active principle of the disinfectant, its concentration, and the initial microbial load (Gleeson et al., 2009).

Although the reduction varied among microorganisms, we observed a decrease in all bacterial groups in POST regardless the treatment. Most environmental mastitis pathogens belong to *Staphylococcus* spp., *Streptococcus* spp., and gram-negative genera (Baumberger et al., 2016). Thus, adopting premilking teat sanitation practices is crucial for minimizing teat exposure to pathogens. By reducing this exposure, the incidence of new intramammary infections can be mitigated, thereby enhancing overall udder health (Baumberger et al., 2016; Ruegg, 2017). However, our findings suggest that an additional application of disinfectant during premilking teat disinfection may not be beneficial when the teats are only lightly dirty.

The mean SCS was 3.53 ± 0.44 for SDA and 3.99 ± 0.44 for DDA, with no significant difference between treatments (*P* = 0.16). The percentage of cows with no subclinical mastitis and new subclinical mastitis is presented in Table 2. No difference in the subclinical mastitis dynamics was observed between treatments, according to the chi-squared test (*P* = 0.18). During the experiment, we diagnosed 20 clinical mastitis cases in 11 cows; 12 in the DDA treatment and 8 in the SDA treatment. In the DDA group, 3 cows had 3 cases of clinical mastitis, whereas 3 other cows had only 1 case each. In the SDA group, 3 cows had 2 cases of clinical mastitis, and 2 cows had only 1 case each. We found no significant difference in clinical mastitis between treatments (*P* = 0.80). Although no differences were observed in subclinical and clinical mastitis between the 2 treatments, this lack of difference could partly be attributed to the low number of cows evaluated, as indicated by the sample size calculation, which reduced the study's power to detect a difference. In addition, the similarity in SCS, subclinical mastitis dynamics, and clinical mastitis occurrences between SDA and DDA treatments is expected, given the similarities in TCS and bacterial counts PRE and POST, as well as the reductions observed between treatments. Morton et al. (2014), evaluating pasture-grazed dairy herds, reported similar clinical mastitis occurrences between cows subjected to no premilking preparation (97 of 1,025 cows) and those receiving premilking preparation with 0.1% iodine disinfectant (98 of 1,029 cows). They attributed these findings to lower rainfall and cleaner, drier teats during the study period, and suggest that under such conditions, routine premilking teat disinfection in pasture-grazed dairy herds may not be economically worthwhile for reducing clinical mastitis cases. However, during periods of heavy teat soiling and increased clinical mastitis incidence, an additional application of disinfectant during premilking disinfection may be beneficial. In confined dairy cows with heavily soiled teats, a double application of disinfectant was beneficial in reducing the TCS, *Staphylococcus* spp. count, and TBC (Niero et al., 2024). Nonetheless, studies on grazing cows in more challenging environments are still needed.

**Table 2:** Chi-squared analysis of the number of observations (N) and percentage of cows with no subclinical mastitis and new subclinical mastitis in cows subjected to a single (SDA) and double (DDA) disinfectant application during premilking teat disinfection^1^

Treatment	N		No subclinical mastitis		New subclinical mastitis	
	Cows	Observations	N	%	N	%
SDA	12	136	80	27.66	18	6.38
DDA	13	146	76	25.89	17	5.67

1 No statistical difference was observed between treatments for any variable (*P* > 0.05).

Our study has some limitations that should be considered. First, the research was conducted on a single dairy farm with a relatively small sample size of 25 cows, due to the limited availability of animals. A small sample size limits the statistical power, and choosing a specific study site (i.e., one farm only) reduces the generalizability. However, the results provide valuable insights for future research. Subsequent studies should include a priori sample size calculations to ensure adequate power for detecting significant effects. Throughout the study period, we evaluated cows at different stages of lactation; however, not all cows were assessed throughout their entire lactation period. Second, the environmental conditions were not particularly challenging for the treatments applied, as the study year was atypical, characterized by drought, with only 3 out of 18 sample collection days having precipitation exceeding 10 mm. Despite these limitations, this study is the first attempt to evaluate the use of a double disinfectant application during premilking teat disinfection in pasture-grazed cows, focusing on the reduction of bacterial count, SCC, and the occurrence of subclinical and clinical mastitis. Future studies with larger sample sizes and conducted in more challenging environments (e.g., muddy conditions leading to soiled teats) are necessary to further investigate the effects of a double disinfectant application during pre-milking teat disinfection on the variables evaluated in this study. Additionally, we encourage future research to explore different combinations of disinfectants and the economic viability of using a double disinfectant application for teat disinfection.

We rejected our hypothesis, as a double application of disinfectant during premilking teat disinfection did not significantly reduce the TCS, bacterial count, SCS, or incidence of subclinical and clinical mastitis compared with a single disinfectant application during premilking teat disinfection under environmental conditions conducive to lightly soiled teats before milking.
